# Feasibility of [^68^Ga]Ga-FAPI-46 PET/CT for detection of nodal and hematogenous spread in high-grade urothelial carcinoma

**DOI:** 10.1007/s00259-022-05761-5

**Published:** 2022-03-24

**Authors:** Lena M. Unterrainer, Simon Lindner, Lennert Eismann, Jozefina Casuscelli, Franz-Josef Gildehaus, Vinh Ngoc Bui, Nathalie L. Albert, Adrien Holzgreve, Leonie Beyer, Andrei Todica, Matthias Brendel, Clemens C. Cyran, Alexander Karl, Christian G. Stief, Stephan T. Ledderose, Marcus Unterrainer, Peter Bartenstein, Vera Wenter, Alexander Kretschmer

**Affiliations:** 1 Department of Nuclear Medicine, University Hospital, LMU Munich, Marchioninistr. 15, 81377 Munich, Germany; 2 Department of Urology, University Hospital, LMU Munich, Munich, Germany; 3 Department of Radiology, University Hospital, LMU Munich, Munich, Germany; 4 Department of Urology, Krankenhaus Barmherzige Brüder, Munich, Germany; 5 Institute of Pathology, LMU Munich, Munich, Germany

**Keywords:** Urothelial carcinoma, Metastatic urothelial carcinoma, Fibroblast activating protein, FAPI, PET/CT imaging

## Abstract

**Background:**

[^68^Ga]Ga-FAPI-46 is a novel positron emission tomography (PET) ligand that targets fibroblast activation protein (FAP) expression as FAP inhibitor (FAPI) and could already show promising results in several tumor entities. It could be demonstrated that an increased FAP expression correlates with tumor aggressivity in urothelial carcinoma (UC). Given the limited value of [^18^F]FDG in UC, [^68^Ga]Ga-FAPI-46 could add diagnostic information in staging and response assessment in UC. We present the first data of [^68^Ga]Ga-FAPI-46 PET imaging in a pilot cohort of UC patients evaluating uptake characteristics in metastases and primary tumors.

**Methods:**

Fifteen patients with UC prior to or after local treatment underwent [^68^Ga]Ga-FAPI-46 PET/CT imaging for detection of metastatic spread. We compared the biodistribution in non-affected organs and tumor uptake of UC lesions by standard uptake value measurements (SUV_mean_ and SUV_max_). Additionally, metastatic sites on PET were compared to its morphological correlate on contrast-enhanced computed tomography (CT).

**Results:**

Overall, 64 tumor sites were detected on PET and/or CT. The highest uptake intensity was noted at the primary site (SUV_max_ 20.8 (range, 8.1–27.8)) followed by lymph node metastases (SUV_max_ 10.6 (range, 4.7–29.1)).

In 4/15 (26.7%) patients there were [^68^Ga]Ga-FAPI-46-positive lesions that were missed on standard routine CT imaging. On the other hand, 2/15 patients had suspicious prominent bipulmonary nodules as well as pelvic lymph nodes previously rated as suspicious for metastatic spread on CT, but without increased FAPI expression; here histopathology excluded malignancy.

**Conclusion:**

[^68^Ga]Ga-FAPI-46 PET shows distinctly elevated uptake in UC lesions. Therefore, the tracer has potential as a promising new biomarker in metastatic UC patients, as [^68^Ga]Ga-FAPI-46 PET might improve detection of metastatic sites compared to CT alone. These findings highly emphasize larger studies investigating FAPI imaging in UC patients.

## Introduction

Urothelial carcinomas (UC) are the fourth most common solid tumors [1–3] and can be located in the lower (bladder and urethra; BC) or the upper (pyelocaliceal cavities and ureter; UTUC) urinary tract [4]. Bladder tumors account for 90–95% of UCs and are the most common urinary tract malignancies. Hereby, UTUC are rare and account for only 5–10% of UC [3], with an estimated annual incidence in Western countries of almost two cases per 100,000 inhabitants [5, 6].

Computed tomography (CT) and magnetic resonance imaging (MRI) are the main diagnostic imaging techniques for staging UC, also in its highly lethal metastatic state [7, 8] even, if there are also new methods for local assessment of urothelial carcinoma like high-resolution micro-ultrasound [9].

Unlike in most other malignancies, the application of ^18^F-fluoro-2-deoxy-2-D-glucose [^18^F]FDG for positron emission tomography (PET) and hybrid PET imaging is of limited diagnostic yield due to low [^18^F]FDG positivity of UC lesions as well of its high renal excretion [10–14]. Therefore, [^18^F]FDG PET is not included by practice guidelines for UC imaging [15].

Fibroblast activation protein (FAP) is highly expressed in the stroma of a variety of human cancers and therefore considered as promising target structure for diagnostic and therapeutic approaches. Calvete et al. could show that FAP expression correlates with tumor staging and aggressive behavior in UC and is expressed by stromal fibroblast adjacent to epithelial tumor cells [16]. Consequently, the use of [^68^Ga]Ga-FAPI-46 PET/CT for pretherapeutic staging and for response assessment might be helpful. Particularly, up to 20% of patients with clinically localized muscle-invasive urothelial carcinoma of the bladder already present with positive lymph node metastases at the time of radical cystectomy [17]. To the best of our knowledge, however, the new FAP inhibitor ligands were not examined in a larger set of urothelial carcinoma patients so far, only cases were published until now [18].

Hence, we hypothesized that a radioligand targeting FAP could be used for pretherapeutic estimation of FAP expression prior to local or systemic therapy in UC, which addresses the unmet need for appropriate pretherapeutic lymph node and distant metastases staging in this tumor entity. In this pilot study, we present the first data on [^68^Ga]Ga-FAPI-46 PET/CT in a population of UC patients to assess the biodistribution of [^68^Ga]Ga-FAPI-46 uptake in metastatic and primary tumor lesions compared to physiological uptake.

## Material and methods

### Patients

We evaluated 15 patients with UC who underwent [^68^Ga]Ga-FAPI-46 PET/CT. All patients gave written consent to undergo [^68^Ga]Ga-FAPI-46 PET/CT according to the regulations of the German Pharmaceuticals Act §13(2b) and were referred for additional imaging by their treating urologist. There was no need for the patients to be fasting. This analysis was performed in compliance with the principles of the Declaration of Helsinki and its subsequent amendments [19] and retrospective analysis of data was approved by the institutional ethics board of the LMU Munich.

### Radiopharmaceuticals/radiosynthesis

Following the regulations of the German Pharmaceuticals Act §13(2b), the labeling of the FAPI tracers was done under the direct responsibility of the applying physician. FAPI-46 was provided by iTheranostics (6162 Bristol Parkway, Culver City, CA 90230, USA).

The radiolabeling of FAPI with ^68^Ga^3+^ obtained from a ^68^Ge/^68^ Ga generator system (GalliaPharm® 50 mCi, Eckert & Ziegler AG, Berlin, Germany) was done by a simple manual synthesis under laminar air flow conditions. Fifty micrograms of FAPI-46 precursor (ABX, Radeberg, Germany) was dissolved in 100 µL Ultrapur water and diluted with 350 µL 0.07 M sodium ascorbate and 400 µL 1.5 M sodium acetate (pH 8.9). Five milliliters of a ^68^Ga^3+^ solution obtained by elution of a ^68^Ge/^68^ Ga generator (Eckert & Ziegler, Berlin, Germany) with 0.1 M HCl was added to the reaction mixture. The mixture was heated at 95 °C for 10 min and diluted with 500 µL 1.5 M sodium acetate and 4 mL water for injection for pH adjustment. After sterile filtration, [^68^Ga]Ga-FAPI-46 was obtained in 98.4 ± 0.57% radiochemical purity. All quality control measurements met the local product release criteria.

### PET/CT acquisition

A mean activity of 210 ± 31 MBq was injected intravenously. Additionally, the patients were premedicated with furosemide (Furosemid-ratiopharm 20 mg/2 mL injection solution, ratiopharm GmbH, Ulm, Germany) for radiation protection and to reduce urinary activity in the renal pelvicalyceal system if no medical contraindication was given [20]. PET was performed using a Biograph 64 PET/CT scanner (Siemens Healthineers, Erlangen, Germany). Approximately 60 min after tracer injection, the PET scan was initiated (2.5 min per bed position). The acquisition time was chosen based on pharmacokinetic data obtained in prior data [21]. For attenuation correction, a low-dose CT without contrast agent was acquired. Images were reconstructed iteratively using TrueX (three iterations, 21 subsets) with Gaussian post-reconstruction smoothing (2 mm full width at half-maximum). Before PET/CT scanning, patients were asked to empty their bladder.

All patients underwent a diagnostic, contrast-enhanced CT prior to [^68^Ga]Ga-FAPI-46 PET/CT for staging purposes as part of the clinical routine within a median time of 15.9 days prior to [^68^Ga]Ga-FAPI-46 imaging.

### Image analysis

Image analysis was performed using a dedicated software package (Hermes Hybrid Viewer, Affinity 1.1.4; Hermes Medical Solutions, Stockholm, Sweden). Biodistribution and tumor uptake in patients were calculated by SUV_max_ and SUV_mean_ measurement.

#### Biodistribution

Organ uptake was evaluated by placing spherical volumes of interest (VOIs) inside the normal, not affected organ parenchyma using a 1-cm diameter VOI for small organs (thyroid, parotid gland, myocardium, adrenal gland) and a 2-cm diameter VOI for muscle, liver, spleen, kidney, fat tissue, aortic lumen (descending aorta), lung, bone (femur), urinary bladder content, uterus, prostate, pancreas body, small intestine, and colon.

#### Tumor sites

In a first step, a visual analysis was performed; tumor lesions/metastatic sites on CT were rated visually as being either PET-positive or PET-negative by two experienced nuclear medicine physicians and two experienced radiologists. For PET quantification of tumor sites, VOIs with a 50% isocontour threshold of the SUV_max_ were automatically generated around tumor lesions with focally increased tracer uptake whenever applicable. In case of close vicinity to areas with high physiological uptake or visually PET-negative lesions, a 1-cm or 2-cm spherical VOI was applied for quantification to exclude high physiological tracer excretion and to ensure reliable quantification of visually negative lesions. Then, tumor-to-liver ratio (TLR), tumor-to-spleen ratio (TSR), and tumor-to-blood pool ratio (as derived from the aorta descendens) (TBR) were calculated by dividing the SUV_max_ and the SUV_mean_ of all tumor lesions by the respective SUV_mean_ of the liver, the spleen, and the arterial blood pool. To ensure a reliable PET quantification, small lung metastases with a SAD ≤ 0.5 cm were not included in the PET quantification analysis but reported as CT findings. In the presence of disseminated hepatic or pulmonary tumor burden, a maximum of five sites in both the PET and CT components was chosen.

### Statistical analysis

Data analysis was performed using Microsoft Excel (Excel 2019, Microsoft, Redmond, WA, USA) and SPSS software (IBM SPSS Statistics 27, Chicago, IL, USA). Descriptive statistics are displayed as median (range) or mean ± standard deviation (SD). Kruskal–Wallis test for unpaired samples was used to determine differences of SUV_mean_, SUV_max_, TLR, TSR, and TBR between different tumor localizations. A two-tailed *p* value < 0.05 was considered statistically significant.

## Results

### Patient characteristics

Five female and ten male patients with a median age of 72.7 (57.6–84.7) years presented for [^68^Ga]Ga-FAPI-46 PET/CT at our department. Two out of 15 (13.3%) patients had a UTUC of the kidney, 1/15 (6.7%) patients had a UTUC of the distal ureter, and 12/15 (80.0%) patients had a UC of the bladder (BC).

Eleven out of 15 (73.3%) patients received a local transurethral tumoral resection of the bladder or upper urinary tract tumor, respectively. One out of 15 (6.7%) patients underwent a nephroureterectomy, 2/15 (13.3%) patients underwent a radical cystectomy, and 1/15 patients (6.7%) underwent a renal biopsy prior to [^68^Ga]Ga-FAPI-46 PET/CT. For further specifications see also Table 1.

**Table 1:** Patient characteristics

No	Age (years)	Sex	Localization	Radical local therapy prior to PET	Systemic therapy prior to PET	Highest pT stage	Tumor grade	Metastatic disease	Localization of metastases	Local tumor burden/distinguishable from urinary activity
1	78	Male	Ureter	n	y	n.a	High-grade	y	Lymph nodes, visceral	y/y
2	84	Male	Bladder	y	n	3a	High-grade	y	Lymph nodes, visceral	n
3	67	Male	Bladder	y	n	Cis	High-grade	y	Lymph nodes, bone	n
4	68	Female	Ureter	y	n	3	High-grade	y	Lymph nodes, visceral	n
5	58	Female	Bladder	n	n	2	High-grade	y	Lymph nodes, visceral, bone	y/n
6	84	Male	Bladder	n	n	2	High-grade	y	Lymph nodes, visceral	y/y
7	73	Female	Bladder	n	n	4a	High-grade	y	Lymph node	y/y
8	66	Male	Bladder	n	n	4a	High-grade	y	Lymph nodes	y/n
9	85	Male	Bladder	n	n	4a	High-grade	y	Lymph nodes, visceral	y/y
10	76	Female	Bladder	n	n	3	High-grade	n	/	y/y
11	66	Male	Bladder	n	n	3	High-grade	n	/	y/n
12	67	Female	Bladder	n	n	3a	High-grade	y	Lymph nodes, visceral, bone	y/n
13	80	Male	Bladder	n	n	2	High-grade	n	/	y/n
14	81	Male	Ureter	n	n	1	High-grade	y	Lymph nodes	y/n
15	60	Male	Bladder	n	n	1	High-grade	n	/	y/n

*n.a.* not available, *y* yes, *n* no

### Biodistribution of [^68^Ga]Ga-FAPI-46

Calculation of SUV_mean_ and SUV_max_ was performed in the static images 1 h post injectionem. Higher SUV_max_ and SUV_mean_ for [^68^Ga]Ga-FAPI-46 were noted in the urinary bladder content, the kidneys, the thyroid, the blood pool, and the prostate whereas lower SUV_max_ and SUV_mean_ values were exemplarily observed in fat tissue, lung, and bone. An extended overview can be found in Table 2.

**Table 2:** Biodistribution (SUV values are displayed as mean ± standard deviation)

Localization	SUV_max_	SUV_mean_
Urinary bladder content	47.1 ± 58.0	40.8 ± 53.1
Kidneys	3.5 ± 1.6	2.6 ± 0.9
Liver	1.3 ± 0.4	0.8 ± 0.4
Spleen	1.3 ± 0.4	0.9 ± 0.2
Uterus (*n* = 4)	4.1 ± 3.7 (near urinary bladder content)	2.7 ± 0.5 (near urinary bladder content)
Prostate (*n* = 6)	2.5 ± 0.6 (near urinary bladder content)	1.9 ± 0.4 (near urinary bladder content)
Aortic lumen (descending)	1.8 ± 0.6	1.4 ± 0.4
Myocardium	1.3 ± 0.3	0.9 ± 0.2
Adrenal glands	1.5 ± 0.8	1.1 ± 0.5
Pancreas body	1.6 ± 0.5	1.2 ± 0.4
Thyroid glands	1.9 ± 0.6	1.5 ± 0.5
Small intestine	1.0 ± 0.4	0.8 ± 0.3
Colon	1.1 ± 0.5	0.9 ± 0.4
Parotid gland	1.3 ± 0.6	0.9 ± 0.5
Muscle	1.5 ± 0.6	1.1 ± 0.4
Fat tissue	0.5 ± 0.2	0.3 ± 0.1
Bone	0.7 ± 0.4	0.5 ± 0.4
Lung	0.5 ± 0.2	0.3 ± 0.1

### Tumor burden

Twelve out of 15 patients (80.0%) prior to planned cystectomy, nephroureterectomy, or systemic therapy had a remaining CT-morphological correlate of the primary tumor (UTUC/BC) after TUR (transurethral resection) even if only 5/12 (41.7%) patients had a FAPI-positive local tumor burden that could be separated by the urinary activity. Consequently, 3/15 (20.0%) patients underwent cystectomy or nephroureterectomy so that no local tumor/remaining tumor was evaluable. Lymph node metastases were observed in 11/15 (73.3%) patients, visceral metastases in 7/15 (46.7%) patients, and bone metastases in 3/15 (20.0%) patients.

Overall, 64 tumor lesions in 15 patients were included; among these 5/64 (7.8%) were local residual tumors at the primary site after transurethral tumor resection or biopsy, 30/64 (46.9%) were lymph node metastases, 18/64 (28.1%) were visceral metastases, and 11/64 (17.2%) were bone metastases.

Overall, there was a median SUV_mean_ of 4.3 (1.1–18.1) and a median SUV_max_ of 8.5 (4.5–29.1). Reporting relative quantitative values, there was a median TLR_mean_ of 5.4 (1.4–23.4) and a median TLR_max_ of 10.5 (3.4–47.1). In relation to the spleen and the blood pool, there was a median TSR_mean_ of 5.5 (1.6–30.2) and a median TSR_max_ of 10.9 (4.9–39.2); median TBR_mean_ was 4.3 (1.3–69.5) and median TBR_max_ was 9.2 (3.0–96.0). An extended overview reporting the uptake parameters in the different tumoral localizations (primary tumor (available in 5/12 patients with a residual primary tumor; 41.7%), lymph node metastases (available in 11/15 patients; 73.3%), visceral metastases (available in 7/15 patients; 46.7%), and bone metastases (available in 3/15 patients, 20%)) can be found in Table 3.

**Table 3:** Comparison of uptake intensities at different tumor localizations (median [range])

Parameter	Local tumor burden	Lymph nodes	Visceral metastases	Bone metastases	Significance
SUV_max_	20.8 (8.1–27.8)	10.6 (4.7–29.1)	7.5 (4.5–13.8)	8.2 (6.0–16.4)	*p* = 0.001
SUV_mean_	13.9 (2.6–18.1)	5.1 (1.1–10.8)	3.1 (1.2–11.0)	4.5 (3.3–6.4)	*p* = 0.001
TLR_max_	24.4 (11.7–47.1)	13.7 (3.6–41.0)	7.9 (4.7–16.2)	9.5 (5.4–23.1)	*p* = 0.053
TLR_mean_	19.9 (3.8–23.4)	6.4 (1.9–15.2)	3.5 (1.4–12.3)	6.6 (2.3–9.9)	*p* = 0.001
TSR_max_	22.9 (13.5–39.2)	12.8 (4.9–36.4)	8.3 (6.3–23.0)	10.2 (7.3–20.5)	*p* = 0.001
TSR_mean_	16.5 (4.3–30.2)	7.1 (1.6–11.5)	3.4 (1.7–18.3)	6.7 (3.0–10.0)	*p* = 0.000
TBR_max_	17.6 (11.3–96.0)	9.9 (3.8–28.0)	7.1 (3.0–14.0)	11.8 (5.7–20.6)	*p* = 0.051
TBR_mean_	9.8 (1.4–69.5)	3.8 (1.3–9.2)	2.6 (1.5–8.2)	5.1 (4.1–6.7)	*p* = 0.006

### Correlation of different tumor localizations

The highest uptake intensity was seen in the FAPI-positive primary tumor sites followed by lymph node metastases, bone metastases, and visceral metastatic sites (e.g., median SUV_max_ 20.8 vs. 10.6 vs. 8.2 vs. 7.5; *p* = 0.001). Evaluating all quantitative parameters, the significantly highest FAPI uptake values were found in the five evaluable tumors at the primary site. For further specifications see also Table 3.

### FAPI-positive metastatic sites not reported on CT imaging

**Fig. 1:**
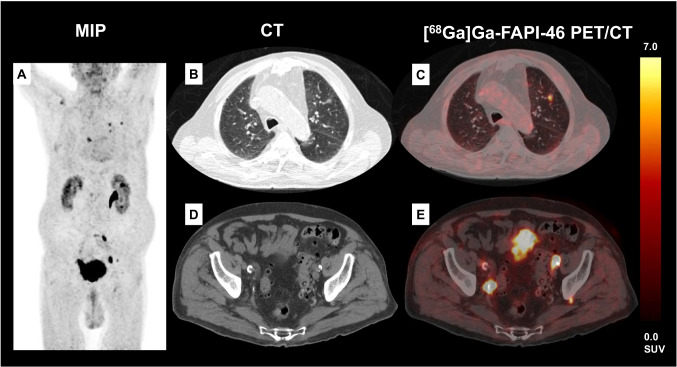
Exemplary patient (**A**: maximum intensity projection (MIP)) with [^68^Ga]Ga-FAPI-46-positive lung metastases of UC in the left upper lobe (**B**, **C**) that were confirmed by histopathology (for histological correlation see also Fig. 2). This patient also presented with a small configuration behind the left acetabular bone (**D**) which could have been missed as lymph node metastases without the increased [^68^Ga]Ga-FAPI-46 expression (**E**)

**Fig. 2:**
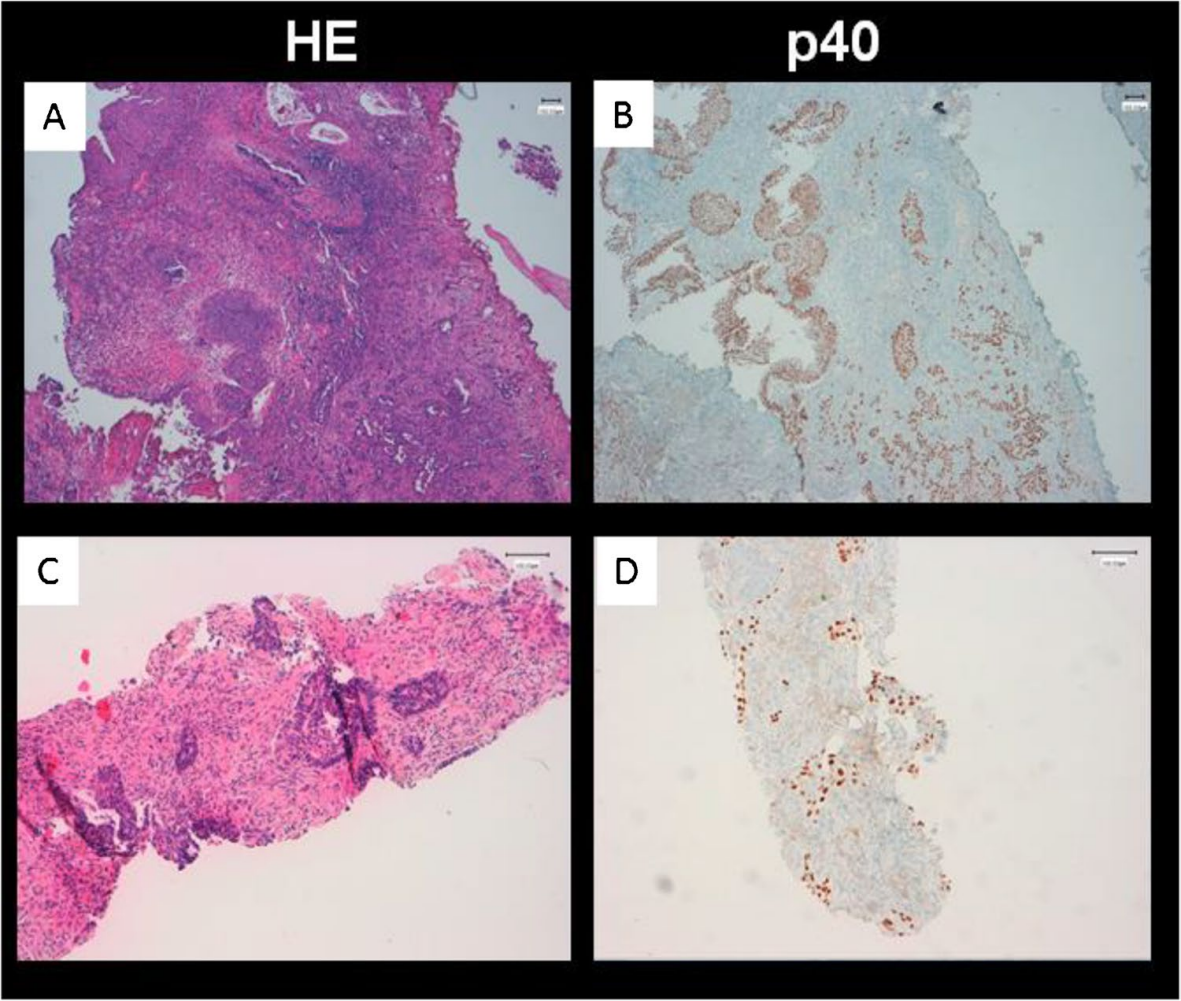
TURB specimen of the same patient as in Fig. 1 shows infiltrates of a partially squamous differentiated high-grade urothelial carcinoma that is p40-positive in immunohistochemical staining (**A**, **B**). Corresponding lung biopsy proves a metastasis of the known bladder carcinoma (**C**, **D**)

Four out of 15 (26.7%) patients showed FAPI-positive lesions that were missed on previous high-dose CT without their increased FAPI uptake: 3/4 patients (75.0%) showed lymph node metastases that were not rated as metastatic sites on the CT component only (patient 1: short-axis diameter (SAD) lymph node 0.8 cm/patient 2: SAD 0.5 cm/patient 3: SAD 1.0 cm) (see also Fig. 1). One out of 4 (25.0%) patients showed FAPI-positive peritoneal spread that was missed in the initial CT staging due to their small size and due to the very sparse amount and one of these patients showed also FAPI-positive lesions in both lungs that were rated as post-inflammatory changes in clinical routine; due to the PET positivity, a histopathological work-up was initiated proving these lesions to be lung metastases of UC (see also Figs. 1 and 2).

### Missing FAPI expression indicates non-tumoral lesions

**Fig. 3:**
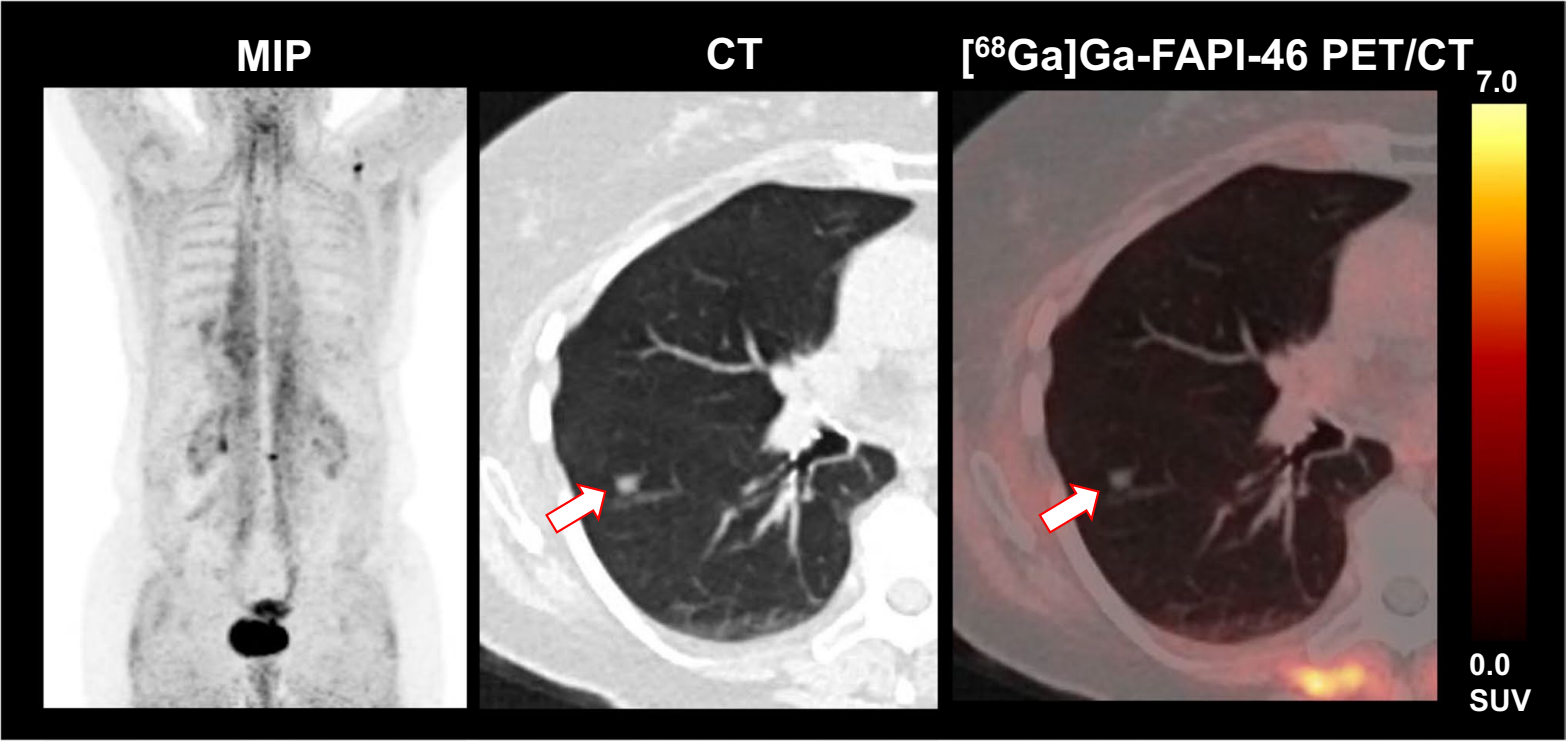
This exemplary patient presented with [^68^Ga]Ga-FAPI-46-negative lung lesions which have been misinterpreted as metastases in CT. However, histopathology excluded metastases and confirmed reactive, inflammatory lung lesions

Two out of 15 (13.3%) patients presented with suspected metastatic side on CT imaging, but without relevant FAPI expression: one patient had FAPI-negative lung lesions with a maximum diameter of 0.8 cm; initially, this was rated as highly suggestive of lung metastasis; CT-guided histopathology, however, excluded malignancy (see also Fig. 3). Another patient showed pathologically enlarged loco-regional lymph nodes which were also rated as highly suggestive of malignancy on CT imaging, whereas on PET imaging, these were also FAPI-negative; histopathology during cystectomy could also exclude lymph node metastases and confirmed non-infiltrated, but reactively enlarged lymph nodes.

### Correlation of histopathology from lymphadenectomy samples with PET results

In 6/10 (60.0%) patients undergoing radical cystectomy after TURB (transurethral resection of the bladder) and PET/CT, a loco-regional lymphadenectomy was performed during the cystectomy procedure; here 3/6 (50.0%) patients showed no [^68^Ga]Ga-FAPI-46-positive lesion which were confirmed histopathologically to be non-UC-related lymph nodes and 2/6 patients showed FAPI**-**positive lymph nodes loco-regionally that were confirmed histopathologically as lymph node metastases of UC. On the other hand, 1/6 patients presented with a histologically proven lymph node metastasis near the external iliac artery and ureter which could not be separated by the urinary activity on PET imaging.

### Non-oncological [^68^Ga]Ga-FAPI-46 uptake

Fifteen non-oncological, FAPI-positive lesions were found in 10/15 (66.7%) patients which could be clearly diagnosed as benign (degenerative, inflammatory) on CT imaging: most of the lesions were degenerative osseous sites (10/15 lesions; 66.6%); in detail one facet joint arthrosis, six omarthrosis, two attachment tendinosis at the shoulder joint, and one osteoarthritis L2/3.

Two out of 15 lesions (13.3%) were non-pathological fractures during healing process, 1/15 (6.7%) lesions was an inflammatory teeth focus, 1/15 (6.7%) lesions was a paragastrical increased [^68^Ga]Ga-FAPI-46 uptake, most likely associated to an inflammatory gastric disease without morphological correlate, and 1/15 (6.7%) lesions was a PET-positive lung dystelectasis.

## Discussion

At the time of radical cystectomy, lymph node metastases can be found in approximately 20% of patients with clinically localized muscle-invasive UC, if no neoadjuvant chemotherapy is performed [22]. However, staging of UCs on a molecular level is not part of the clinical routine especially due to the limited value of [^18^F]FDG in UC patients [10, 11]. Tackling this unmet clinical need for improved molecular imaging, we hypothesized that the new ligand [^68^Ga]Ga-FAPI-46 might have an advantage in the staging of clinically localized UC for evaluating lymph node status as well as for excluding distant metastases, as this might impact the clinical management of UC patients at early stages.

Firstly, we evaluated the physiological distribution of [^68^Ga]Ga-FAPI-46 as well as the tracer uptake in tumoral lesions (SUV_mean_, SUV_max_) and compared the tumoral uptake to the physiological uptake of liver, spleen, and blood pool. The highest physiological accumulation of [^68^Ga]Ga-FAPI-46 was detected inside the urinary bladder content and the kidneys; lower tracer uptakes were exemplarily seen in colon and lung. Our results are in line with already published biodistribution studies [21, 23–25]. Also, the relevant portion of non-oncological [^68^Ga]Ga-FAPI-46 uptake matches to the results in recent studies, allowing the use of [^68^Ga]Ga-FAPI-46 also for evaluating benign diseases like inflammation or degenerative diseases [26–28]. However, a deepened knowledge of the physiological tracer distribution and non-malignant findings on FAPI imaging is mandatory, as recently published [29].

Assessing the uptake characteristics of tumor sites, our results demonstrated a high rate of PET-positive lesions with highest uptake characteristics in the primary tumor site. We observed that in a considerable proportion of patients in the area of the CT-morphologically delineable tumor, the local FAPI expression could not be distinguished from the FAPI expression of the urinary tract: this observation suggests that [^68^Ga]Ga-FAPI-46 might not have an advantage over conventional imaging such as MRI or a cystoscopy for evaluating local tumoral extension in UC patients.

Given the current cohort of patients, a part of the patients, however, had a local tumor-debulking surgery prior to [^68^Ga]Ga-FAPI-46 PET/CT for histological assessment, which suggests low [^68^Ga]Ga-FAPI-46 expression due to the absence of a clear macroscopically definable tumor burden, at least partly. On the other hand, other FAPI ligands such as ^18^F-labeled compounds might have a lower urinary excretion and could be superior for local staging compared to [^68^Ga]Ga-FAPI-46 [24]; here further studies evaluating other FAPI ligands are necessary.

Nonetheless, it should be mentioned that the local tumor burden is often not the decisive point regarding a curative therapy such as a cystectomy: rather, the presence of distant metastases decides on the guideline-compliant therapy [15].

Regarding metastatic sites, there was a high rate of congruent findings of CT and PET, where pathologically enlarged lymph nodes also were FAPI-positive on PET, which underlines the “true positive” aspects of FAPI-positive lymph nodes in concordance with CT imaging. Interestingly, in 4/15 patients, however, tumoral lesions were classified as malignant and UC-associated based on their FAPI positivity only, where CT was not rated as suspicious: especially in patients with muscle-invasive UC, who are planned for cystectomy and who are treated guideline-appropriate only in case of absence of distant metastases, [^68^Ga]Ga-FAPI-46 PET could have a significant added value in terms of proper clinical management.

On the other hand, two patients showed FAPI-negative, CT-morphologically suspicious pelvic lymph nodes as well as intrapulmonary lesions. Regarding CT only, these lesions were rated highly suspicious for UC metastases. However, histopathology classified them as non-tumor-associated sites. These observations might lead to the assumption that [^68^Ga]Ga-FAPI-46 provides a high rate of single-lesion specificity. Consequently, we hypothesize that [^68^Ga]Ga-FAPI-46 PET/CT help to properly “down-stage” patients with falsely rated suspicious findings on CT only, where, e.g., reactively enlarged lymph nodes or post-infectious pulmonary findings might mimic metastatic spread in UC patients. Here, the use of advanced molecular imaging such as [^68^Ga]Ga-FAPI-46 might lead to the avoidance of unnecessary biopsies, e.g., in case of clearly missing FAP expression.

In correlation with the histology findings, we observed a high concordance of FAPI positivity and malignancy in lymph nodes and vice versa; in five patients undergoing radical cystectomy with concomitant lymphadenectomy, the FAPI positivity was predictive for the presence of lymph node metastases and vice versa, so that one might hypothesize that the addition of PET imaging might significantly improve the nodal staging prior to further therapies. Especially in patients with lymphadenectomy in addition to a cystectomy, which itself is a radical surgical intervention, a preoperative FAPI PET/CT could possibly lead to an omission of a supplementary lymphadenectomy—if the FAPI PET/CT was negative. However, prospective studies correlating the results of the FAPI PET/CT (FAPI-positive/FAPI-negative lymph nodes) directly with the histopathological results are necessary to further support this hypothesis.

Beyond staging purposes, it seems obvious to speculate that [^68^Ga]Ga-FAPI-46 could be used to monitor systemic therapy in metastatic disease stage, while a decrease in [^68^Ga]Ga-FAPI-46 expression might possibly be consistent with a response to therapy.

Given the potential labeling of [^68^Ga]Ga-FAPI-46 with a DOTA-chelator [30, 31], also a theranostic approach might be thinkable, so that [^68^Ga]Ga-FAPI-46 could also be applied for the assessment of uptake intensity before a potential therapy with, e.g., [^177^Lu]Lu-FAPI-46-labeled ligands in UC patients that have no other therapy options left [32].

A major limitation of this pilot analysis is the retrospective design as well as the small number of patients; especially, we could only include 3 patients with UTUC: here further studies are needed evaluating a bigger population of urothelial carcinomas of the upper urinary tract to identify the value of [^68^Ga]Ga-FAPI-46 PET for this urothelial carcinoma subtype concerning evaluation of N- and M-staging. In addition, 20% of our patients did not have detectable residual local tumor burden. Consequently, [^68^Ga]Ga-FAPI-46 uptake at the primary tumor side could not be evaluated in a minor portion of the included cases. Therefore, further studies are needed which correlate [^68^Ga]Ga-FAPI-46 uptake with the different UC histopathological subtypes and molecular genetic features, which targets the potential capacity of detection of lymph node metastases due to [^68^Ga]Ga-FAPI-46 PET/CT. Furthermore, in vivo- as well as in vitro-autoradiography binding studies with immunohistochemical correlations as well as preclinical studies are needed to exactly determine in direct spatial correlation the respective binding in UC specimens.

## Conclusion

[^68^Ga]Ga-FAPI-46 PET shows distinctly elevated uptake in UC lesions. [^68^Ga]Ga-FAPI-46 PET might be used for the detection of nodal and hematogenous sites prior to systemic treatment and, hence, might influence the patient’s management by detection, but also by exclusion of distant spread in UC patients.

## References

[1] Sjödahl G (2012). A molecular taxonomy for urothelial carcinoma. Clin Cancer Res.

[2] Yafi FA, North S, Kassouf W (2011). First-and second-line therapy for metastatic urothelial carcinoma of the bladder. Curr Oncol.

[3] Sung H (2021). Global cancer statistics 2020: GLOBOCAN estimates of incidence and mortality worldwide for 36 cancers in 185 countries. CA Cancer J Clin.

[4] Green DA (2013). Urothelial carcinoma of the bladder and the upper tract: disparate twins. J Urol.

[5] Rouprêt M (2021). European Association of Urology guidelines on upper urinary tract urothelial carcinoma: 2020 update. Eur Urol.

[6] Zattoni F (2019). 18F-FDG PET/CT and urothelial carcinoma: impact on management and prognosis—a multicenter retrospective study. Cancers.

[7] Bellmunt J (2017). Pembrolizumab as second-line therapy for advanced urothelial carcinoma. N Engl J Med.

[8] Campbell SP (2018). Low levels of PSMA expression limit the utility of 18 F-DCFPyL PET/CT for imaging urothelial carcinoma. Ann Nucl Med.

[9] Saita A (2020). Assessing the feasibility and accuracy of high-resolution microultrasound imaging for bladder cancer detection and staging. Eur Urol.

[10] Zhang H (2015). Diagnostic value of [18 F] FDG-PET and PET/CT in urinary bladder cancer: a meta-analysis. Tumor Biology.

[11] Soubra A (2016). The diagnostic accuracy of 18F-fluorodeoxyglucose positron emission tomography and computed tomography in staging bladder cancer: a single-institution study and a systematic review with meta-analysis. World J Urol.

[12] Pichler R (2017). Pelvic lymph node staging by combined 18F-FDG-PET/CT imaging in bladder cancer prior to radical cystectomy. Clin Genitourin Cancer.

[13] Vind-Kezunovic S (2019). Detection of lymph node metastasis in patients with bladder cancer using maximum standardised uptake value and 18F-fluorodeoxyglucose positron emission tomography/computed tomography: results from a high-volume centre including long-term follow-up. Eur Urol Focus.

[14] Lakhani A (2017). FDG PET/CT pitfalls in gynecologic and genitourinary oncologic imaging. Radiographics.

[15] Witjes JA, Compérat E, Cowan NC, De Santis M, Gakis G, Lebret T, Sherif A. EAU guidelines on muscle-invasive and metastatic bladder cancer: summary of the 2013 guidelines. Eur Urol. 2014;65(4):778–792. 10.1016/j.eururo.2013.11.046 24373477

[16] Calvete J (2019). The coexpression of fibroblast activation protein (FAP) and basal-type markers (CK 5/6 and CD44) predicts prognosis in high-grade invasive urothelial carcinoma of the bladder. Hum Pathol.

[17] Mertens L, et al. Occult lymph node metastases in patients with muscle invasive bladder cancer: incidence after neoadjuvant chemotherapy and cystectomy versus cystectomy alone. BJU Int. 2013. 10.1111/bju.12447 24053889

[18] Dendl K, Finck R, Giesel FL, Kratochwil C, Lindner T, Mier W, Koerber SA. FAP imaging in rare cancer entities—first clinical experience in a broad spectrum of malignancies. Eur J Nucl Med Mol Imaging. 2022;49(2):721-731. 10.1007/s00259-021-05488-9 PMC8803688 34342669

[19] Assembly WMAG (2004). World Medical Association Declaration of Helsinki: ethical principles for medical research involving human subjects. J Int bioeth.

[20] d'Amico A (2014). Effect of furosemide administration before F-18 fluorodeoxyglucose positron emission tomography/computed tomography on urine radioactivity and detection of uterine cervical cancer. Nucl Med Rev.

[21] Giesel FL (2019). 68Ga-FAPI PET/CT: biodistribution and preliminary dosimetry estimate of 2 DOTA-containing FAP-targeting agents in patients with various cancers. J Nucl Med.

[22] Vazina A (2004). Stage specific lymph node metastasis mapping in radical cystectomy specimens. J Urol.

[23] Meyer C (2020). Radiation dosimetry and biodistribution of ^68^Ga-FAPI-46 PET imaging in cancer patients. J Nucl Med.

[24] Giesel FL (2021). FAPI-74 PET/CT using either 18F-AlF or cold-kit 68Ga labeling: biodistribution, radiation dosimetry, and tumor delineation in lung cancer patients. J Nucl Med.

[25] Syed M (2020). Fibroblast activation protein inhibitor (FAPI) PET for diagnostics and advanced targeted radiotherapy in head and neck cancers. Eur J Nucl Med Mol Imaging.

[26] Lan L, et al. The potential utility of [68 Ga] Ga-DOTA-FAPI-04 as a novel broad-spectrum oncological and non-oncological imaging agent—comparison with [18F] FDG. Eur J Nucl Med Mol Imaging. 2021: 1–17. 10.1007/s00259-021-05522-w 34410435

[27] Dendl K (2021). FAP and FAPI-PET/CT malignant and non-malignant diseases: a perfect symbiosis?. Cancers.

[28] Qiu L, et al. The potential utility of 68Ga-FAPI-04 as a novel broad-spectrum tumor and inflammatory imaging agent-comparison with 18F-FDG. 2021.

[29] Kessler L, et al. Pitfalls and common findings in 68Ga-FAPI-PET–a pictorial analysis. J Nucl Med. 2021. 10.2967/jnumed.121.262808 PMC9157730 34620730

[30] Lindner T (2018). Development of quinoline-based theranostic ligands for the targeting of fibroblast activation protein. J Nucl Med.

[31] Altmann A (2021). Ligand engineering for theranostic applications. Curr Opin Chem Biol.

[32] Ferdinandus J, et al. Initial clinical experience with 90Y-FAPI-46 radioligand therapy for advanced stage solid tumors: a case series of nine patients. J Nucl Med. 2021. 10.2967/jnumed.121.262468 PMC9051597 34385340

